# A Micro-Scale Analytical Method for Determining Glycogen Turnover by NMR and FTMS

**DOI:** 10.3390/metabo12080760

**Published:** 2022-08-18

**Authors:** Timothy L. Scott, Juan Zhu, Teresa A. Cassel, Sara Vicente-Muñoz, Penghui Lin, Richard M. Higashi, Andrew N. Lane, Teresa W.-M. Fan

**Affiliations:** 1 Center for Environmental and Systems Biochemistry and Markey Cancer Center, University of Kentucky, Lexington, KY 40506, USA; 2 Center for Environmental and Systems Biochemistry, University of Kentucky, Lexington, KY 40506, USA; 3 Center for Environmental and Systems Biochemistry, Department Toxicology & Cancer Biology and Markey Cancer Center, University of Kentucky, Lexington, KY 40506, USA

**Keywords:** glycogen turnover, ^13^C_6_-glucose, stable isotope resolved metabolomics (SIRM), microwave-assisted hydrolysis

## Abstract

Glycogen is a readily deployed intracellular energy storage macromolecule composed of branched chains of glucose anchored to the protein glycogenin. Although glycogen primarily occurs in the liver and muscle, it is found in most tissues, and its metabolism has been shown to be important in cancers and immune cells. Robust analysis of glycogen turnover requires stable isotope tracing plus a reliable means of quantifying total and labeled glycogen derived from precursors such as ^13^C_6_-glucose. Current methods for analyzing glycogen are time- and sample-consuming, at best semi-quantitative, and unable to measure stable isotope enrichment. Here we describe a microscale method for quantifying both intact and acid-hydrolyzed glycogen by ultra-high-resolution Fourier transform mass spectrometric (UHR-FTMS) and/or NMR analysis in stable isotope resolved metabolomics (SIRM) studies. Polar metabolites, including intact glycogen and their ^13^C positional isotopomer distributions, are first measured in crude biological extracts by high resolution NMR, followed by rapid and efficient acid hydrolysis to glucose under N_2_ in a focused beam microwave reactor, with subsequent analysis by UHR-FTMS and/or NMR. We optimized the microwave digestion time, temperature, and oxygen purging in terms of recovery versus degradation and found 10 min at 110–115 °C to give >90% recovery. The method was applied to track the fate of ^13^C_6_-glucose in primary human lung BEAS-2B cells, human macrophages, murine liver and patient-derived tumor xenograft (PDTX) in vivo, and the fate of ^2^H_7_-glucose in ex vivo lung organotypic tissue cultures of a lung cancer patient. We measured the incorporation of ^13^C_6_-glucose into glycogen and its metabolic intermediates, UDP-Glucose and glucose-1-phosphate, to demonstrate the utility of the method in tracing glycogen turnover in cells and tissues. The method offers a quantitative, sensitive, and convenient means to analyze glycogen turnover in mg amounts of complex biological materials.

## 1. Introduction

Glycogen is a major macromolecular storage form of glucose in mammals, which can be rapidly mobilized to provide energy (3 ATP/glucose oxidized to pyruvate) via glycogenolysis and to serve as a carbon source for anabolic metabolism [1,2]. It is abundant in liver, skeletal muscle, and kidney, but is also found in many other tissues, including brain, lung, and some solid tumors [3,4]. Dysregulation of glycogen metabolism results in many human diseases including several forms of storage diseases (e.g., Lafora disease) and may contribute to the development of diabetes and some cancers [5,6].

**Figure 1:**
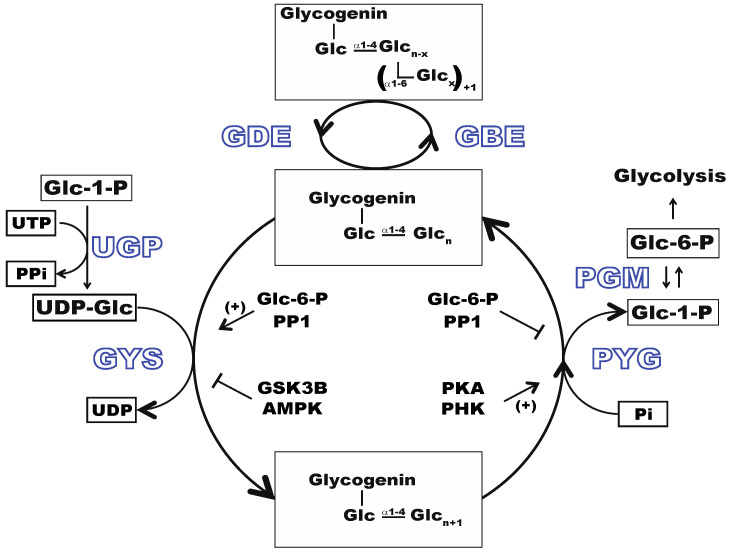
Glycogen metabolism pathway. Glucose-6-phosphate (Glc-6-P) is converted to Glucose-1-phosphate (Glc-1-P) by phosphoglucomutase (PGM). Glc-1-P is then converted to UDP-glucose (UDP-Glc) by UDP-glucose pyrophosphorylase (UGP). Glycogen is synthesized by successive addition of Glc to existing glycogen, catalyzed by glycogen synthase (GYS) and branching enzyme (GBE). The breakdown is catalyzed by the debranching enzyme (GDE) and phosphorylase (GYB). The glycogen turnover enzymes are inversely regulated by phosphorylation/dephosphorylation and by allosteric interactions.

Glycogen is a highly branched polymer of glucose, comprising linear chains linked via α1-4 glycosidic bonds, with α1-6 glycosidic bonded branches approximately every 8–12 residues [7]. The branched core particles are assembled on an autoglucosylating initiator protein, glycogenin [8]. Subsequent glycogen synthesis on an existing structure is catalyzed by two enzymes, glycogen synthase (GYS) and the branching enzyme (GBE). Glycogen breakdown requires two other enzymes, phosphorylase (PYG) and debranching enzyme (GDE). The synthesis and degradation of glycogen is highly regulated [9], requiring the coordinated activity of these enzymes that are controlled at multiple levels including post-translational and allosteric modifications (Figure 1). GYS is activated allosterically by glucose-6-phosphate (Glc-6-P) and post-translationally by de-phosphorylation via protein phosphatase 1 (PPI). Conversely, GYS is inactivated by phosphorylation by kinases, such as Glycogen Synthase Kinase 3β (GSK3B) and/or AMPK [10]. PYG is activated by phosphorylation, though such activation is also dependent on allosteric interactions with Glc-6-P and AMP, which varies with the different isoforms of the enzyme [11]. GYS and PYG isoform expression is tissue dependent with GYS1 and PYGM expressed in the skeletal muscle, GYS2 and PYGL in the liver, and GYS1 and PYGB in the brain, heart, and liver. They are also found to be highly expressed in several cancers, including breast, kidney, uterus, bladder, ovary, skin, and brain tumors [12].

We and others have analyzed intact glycogen and its synthesis using the tracer ^13^C_6_-glucose with nuclear magnetic resonance (NMR) in cancer cells and tissues [13,14] as well as liver [15,16]. However, due to the macromolecular nature of glycogen, the total glycogen content may not be accurately determined by NMR, as the immobile parts of the glycogen structure may not be NMR-detectable. Multiple methods for glycogen analysis have been developed including several microscopy-based approaches and enzymatic or chemical means that break down glycogen for analysis. Common microscopy methods include (i) electron microscopy [17], which is qualitative; (ii) antibody-based histochemistry [18,19,20,21], and (iii) histochemistry using the periodic acid oxidation and Schiff reaction (PAS) [22], which has low sensitivity and is non-specific for hexoses. Live cell/tissue imaging techniques utilizing fluorescent markers or radioisotopes have also been developed [23,24]. These methods have low resolution and sample throughput as well as being at best semi-quantitative. More quantitative methods for glycogen analysis are available including enzymatic or acid digestion to release glucose, followed by glucose analysis via enzyme assays or GC-MS after derivatization [25,26]. However, these methods are not designed for high throughput or small samples. Importantly, of the methods described, only the GC-MS method can provide isotopic label (e.g., ^13^C) distribution of hydrolyzed glycogen, but cannot resolve different stable isotopes (e.g., ^13^C, ^2^H) in stable isotope tracer studies to inform on the rates of de novo synthesis and degradation of glycogen. To overcome these limitations, we have developed a microscale method to determine both intact glycogen and its constituent glucose after acid hydrolysis using ultra high-resolution Fourier transform mass spectrometric (UHR-FTMS) and NMR. Rapid hydrolysis was achieved with microwave-assisted digestion, which we have previously shown for protein turnover analysis [27]. The method is rapid, quantitative, with minimal destruction of released monomers, and compatible with our stable isotope resolved metabolomics (SIRM) workflow [14,28,29,30]. We have applied the method to track ^13^C_6_-glucose and ^2^H_7_-glucose metabolism into glycogen and pathway intermediates in cells, tumor tissues, and live animals including mice bearing PDTX.

## 2. Materials and Methods

Glycogen from bovine liver, type IX (G0885-1G, lot # 100M7005V), was purchased from Sigma-Aldrich (St. Louis, MO, USA), trace metal grade HCl (A508-4) from Fisher Scientific (Fair Lawn, NJ, USA), Dulbecco’s modified eagle media (DMEM) (D5030) from Sigma (St. Louis, MO, USA), Fetal Bovine Serum (FBS) (SH30071.03) from HyClone (Logan, UT, USA), D-(+)-Glucose (194672) from MP Biomedicals (Santa Ana, CA, USA), L-glutamine (BP379-100) from Fisher Scientific (Fair Lawn, NJ, USA). Pen-Strep 100X solution (SV30010) from Fisher Scientific (Fair Lawn, NJ, USA).

^13^C_6_-glucose (CLM-1396-MPT-PK), ^2^H_7_-glucose (DLM-2062-PK), ^13^C_5_-glutamine (NLM-1822H-PK), ^15^N_2_-tryptophan (NLM-800-PK) were purchased from CIL (Tewksbury, MA, USA), ^13^C_6_H_5_D_7_O_6_ (608238). from Sigma (St. Louis, MO, USA).

Immortalized bronchial airway cells BEAS-2B (CRL-9609) were purchased from ATCC (Manassas, VA, USA).

The NOD.Cg-*Prkdc^scid^*/*l2rg^tm1Wjl^*/*SzJ* (NSG) and C57BL/6J mice were from an in-house breeding colony originally purchased from the Jackson Laboratory (Bar Harbor, ME, USA) Stock No. 005557 and 000664, respectively. Basal Mix for liquid diet (TD.150344.PWD) was purchased from Envigo (Indianapolis, IN, USA). All other reagents were of analytical or molecular biology grade.

### 2.1. ^13^C_6_-Glucose Treatments of Cell Cultures

#### 2.1.1. BEAS-2B Cells

Human bronchial epithelial BEAS-2B cells were grown on 10 cm dishes at 37 °C in an atmosphere of 20% O_2_/5% CO_2_ at a relative humidity of >90% to a confluency of 70%. Cell culture medium consisted of DMEM supplemented with 10% FBS, 0.45% glucose, 2 mM glutamine, and 1X penicillin-streptomycin antibiotics. The medium was changed 24 h prior to the cell harvest to glucose-free DMEM supplemented with unlabeled or ^13^C_6_-glucose plus glutamine, antibiotics, and FBS. The cells were then harvested by washing and quenching as described below. The glycogen standard was added just prior to quenching.

#### 2.1.2. Human Peripheral Blood Monocytes (PBMC)-Derived Macrophages

Monocytes were isolated from whole human peripheral blood of an age 65 male volunteer using the RosetteSep™ human monocyte enrichment cocktail kit (StemCell). Monocytes were differentiated in 6-well plates (Nunclon, Thermo Fisher, Waltham, MA, USA) for 8 days in monocyte differentiation medium (MDM) containing DMEM, 10% FBS, 10 mM glucose, 2 mM Gln, 1X antibiotic-antimycotic, and 50 ng/mL CSF-1 at 37 °C/5% CO_2_ at a density of 3–5 × 10^6^ cells per well before polarization. 

Cells were then placed in the MDM with glucose replaced by ^13^C_6_-glucose for 24 h. Metabolites were extracted and analyzed by NMR for glycogen as described below.

### 2.2. Liquid Diet Feeding of [^13^C_6_]-Glucose in Mice

We determined the glycogen content and ^13^C labeling in tissues of C57BL/6J and NOD.Cg-Prkdc^scid^Il2rg^tm1Wjl^/SzJ (NSG) mice fed a ^13^C_6_-glucose enriched liquid diet as described previously [16]. Lung cancer tissues for the PDTX were procured from patients undergoing surgery at the University of Kentucky and subcutaneously implanted into the shoulders of immune compromised NSG mice. The xenografts were allowed to grow to approximately 1 cm in diameter before excision. Tumor and/or liver tissues were excised and metabolically quenched (see below) within approximately 3 min of euthanizing. All experiments were approved by the University of Kentucky IRB and IACUC (see below). 

### 2.3. Multi-Tracer Treatments of Ex Vivo Organotypic Cultures (OTC) of Human Lung Tissues 

Cancerous (CA) and surrounding non-cancerous (NC) lung tissues were freshly collected from a non-small cell lung cancer (NSCLC) patient in the operating room. The pair of tissues were placed in PBS, thinly sliced using a Krumdieck (Alabama Research & Development, Munford AL) microtome, and incubated in ^2^H_7_-Glucose, ^13^C_5_-glutamine, and ^15^N_2_-tryptophan glucose/Gln/Trp-free DMEM medium plus 10% dialyzed fetal bovine serum (FBS), 1X penicillin/streptomycin for 48 h at 37 °C/5% CO_2_ with gentle rocking to facilitate nutrient exchange and waste product mixing [31,32]. Culture media were sampled at 0, 24, and 48 h of incubation. Tissue slices were then quickly washed and processed as described below.

### 2.4. Metabolic Quenching and Extraction of Metabolites

Bulk human tissues were immediately flash-frozen at the operating room to quench metabolism. Cells in plates or mouse tissues were quickly rinsed twice in cold PBS and once in Nanopure water. Cells were then metabolically quenched directly in cold acetonitrile while mouse tissues were quenched in liquid N_2_. Human OTC slices were rinsed as mouse tissues, vacuum-suctioned to remove excess liquid, weighed on a 4-place balance for wet weight, and flash-frozen in liquid N_2_. Frozen tissues were stored at −80 °C until pulverization to a fine powder under liquid N_2_ and extracted for polar metabolites as previously described [16,33]. 

Free glucose and glycogen in polar extracts was analyzed by 1D and 2D NMR prior to acid hydrolysis. Total NMR-visible glycogen was determined by integrating the resonance at 5.41 ppm, which corresponds to the H1α resonance of glucose residues in glycogen [34,35]. Free glucose and/or Glc-6-P was analyzed for both the alpha and beta conformers using the ^1^H resonances at 5.22 and 4.64 ppm, respectively, which are in a ratio of 1:2. The NMR spectra were also used for the assignment and quantification of other metabolites [35,36].

### 2.5. Acid Hydrolysis

After NMR analysis, the sample in a 1.7 mm NMR tube was recovered quantitatively into a 0.5 mL microcentrifuge tube by inverting the NMR tube attached to a piece of silicon tubing into the tube and centrifuging the assembly at 500 g. The recovered sample was then transferred to a pre-tared Target Glass flat bottom Micro-Sert (National NSC C4011-631, Thermo Scientific). The NMR tube was washed twice with 50 µL of Milli-Q water with the washes recovered in the same manner and pooled with the initial sample. The 0.5 mL recovery tube was then washed with an additional 50 µL and combined with the previous recovered sample. The pooled samples were then lyophilized prior to acid hydrolysis.

The samples in the micro-serts were reconstituted with 50 µL of 1 N trace metal free HCl (Fisher). The micro-serts were then vortexed briefly and centrifuged to ensure that the solution was at the bottom of the micro-sert. The pre-hydrolysis mass was recorded before loading the micro-sert into a CEM (CEM Corp, Matthews, NC, USA) 10 mL focused microwave reaction vessel with 300 µL 1 N HCl added to the outside of the micro-sert to prevent sample evaporation. The reaction vessel was sealed with the CEM pressure transducer septum cap and nitrogen purged via syringe needles for 20 min with a flow rate between 0.1 to 0.5 LPM.

The acid hydrolysis reaction was run in a CEM Discover SP focused microwave digester equipped with an Explorer autosampler (CEM Corp, Matthews, NC, USA) at 75 W, a pressure maximum of 200 psi, and variable temperatures and time for method development. After the reaction, the vessels were centrifuged for 5 min at 500× *g* and allowed to cool at 4 °C for up to 10 min to avoid HCl spurts when opening the cap. The micro-serts were then removed from the reaction vessels, and the exterior blotted dry with paper towel, then weighed to determine the final volume. Next, the samples were immediately analyzed by UHR-FTMS or transferred quantitatively to a 1.5 mL microcentrifuge tube with 3 washes of the micro-serts at 100 µL 18 MOhm water each and lyophilized before NMR analysis.

### 2.6. NMR Spectroscopy

Cell and tissue polar extracts were reconstituted in 50 µL ^2^H_2_O (>99.9%, Cambridge Isotope Laboratories, MA, USA) containing respectively 0.5 mmole/L of ^2^H_6_-2,2-dimethyl-2-silapentane-5-sulfonate (DSS) and DSS + 1 mM ^2^H_16_-EDTA before loading into 1.7 mm NMR tubes. One-dimensional (1D) NMR spectra were recorded at 288 K on a 14.1 T Agilent DD2 spectrometer (Agilent Technologies, Santa Clara, CA, USA) equipped with a 3 mm inverse triple resonance HCN cold probe. For each sample, 1D ^1^H and ^1^H{^13^C} HSQC spectra were recorded. 1D ^1^H spectra were acquired with an acquisition time of 2 s and a relaxation delay of 4 s, during which the residual HOD resonance was irradiated. ^1^H spectra were processed with one zero-filling and 1 Hz line-broadening exponential function. 1D ^1^H{^13^C} HSQC spectra were recorded with an acquisition time of 0.25 s and a relaxation delay of 1.75 s. The spectra were processed by zero-filling twice, apodization with an unshifted Gaussian function, and a line exponential broadening of 4 Hz.

2D NMR experiments were recorded on a Bruker Avance III spectrometer (Bruker BioSpin, Billerica, MA, USA) operating at 16.45 T equipped with a 1.7 mm cryogenically cooled HCNP inverse quad probe. 2D ^1^H-^1^H TOtal Correlation SpectroscopY (TOCSY) spectra were acquired with an isotropic mixing time of 50 ms at a B_1_ strength of 10 kHz, with acquisition times of 1 s in t_2_ and 0.064 s in t_1_. The spectra were processed with 1 linear prediction and 1 zero-filling in t_1_, with an unshifted Gaussian apodization function and 1 Hz line broadening exponential in both dimensions. DSS-^2^H_6_ was used as an external standard for both chemical shift referencing and absolute quantification. NMR spectra were analyzed using MNova (MestReNova v. 9, Santiago de Compostela, Spain) or Topspin 3.5 (Bruker BioSpin, Billerica, MA, USA) and the glucose/glycogen value obtained was normalized to mg of protein. Standard chemical shifts for compound identification were taken from [35,36].

The fraction ^13^C, F was calculated as:

F = I(^13^C)/[I(^13^C) + I(^12^C)]

where I(^13^C) is the intensity of the ^13^C satellite and I(^12^C) is the intensity of the unlabeled resonance.

### 2.7. UHR-FTMS Analysis

UHR-FTMS analysis was carried out using the Tribrid Fusion Orbitrap (Thermo Scientific, San Jose, CA, USA), interfaced with an Advion Triversa Nanomate (Advion Biosciences, Ithaca, NY, USA). Each sample was serial diluted 10–1000 times in 0.1% PBS or 18 MOhm water. The spray solution was a 1:1:10 mixture of diluted sample, internal standard, acetonitrile, which was prepared fresh before sample introduction into the FTMS via the Nanomate operated at 1.5 kV and 0.5 psi head pressure in positive ion mode. The FTMS data were acquired using the maximum resolution setting of 500,000 or 1,000,000, with the maximal ion time for the automatic gain control (AGC) at 100 ms, 5 micro scans per stored spectrum, and >5 min of spectral collection over an *m*/*z* range 100–1000 selected using quadrupole isolation. MS^1^ was used throughout the study to analyze for all glucose isotopologues from any stable isotope tracer (in this study, ^13^C and ^2^H) and hydrolysis breakdown products at once. The external standard, ^13^C_6_-Glucose, was added for unlabeled experiments, or ^13^C_6_,^2^H_7_-Glucose for labeled experiments, at a level approximately within 10-fold of the tallest detected glucose peak. For data analysis, a composite spectrum was used for each sample by averaging spectra over the entire acquisition time [27].

### 2.8. Glycogen Calculation

Glycogen was quantified using the following equations.

Post hydrolysis glucose − pre hydrolysis glucose = net glucose

Glycogen in µmoles/mg protein = net glucose/6.17 µmoles glucose per mg glycogen/hydrolysis efficiency from glycogen standard/mg cell proteins.

Glucose equivalent per mg glycogen was calculated by subtracting H_2_O (18.01 g/mole) from glucose (180.06 g/mole) to give 162.05 g/mole or 6.17 µmoles glucose/mg glycogen.

### 2.9. Statistical Analyses

Means and standard errors were calculated, and where comparisons were made, the unpaired *t*-test was used, with correction for false discovery rate.

## 3. Results

### 3.1. Optimization of Glycogen Hydrolysis

Previous hydrolysis-based glycogen determination methods required large quantities of starting material (0.1–100 g) and long reaction times [37,38,39,40]. We utilized a microwave-assisted method to efficiently hydrolyze nanogram to microgram amounts of glycogen extracted from mg of samples from various sources. The same extracts are also compatible for metabolomics analysis, i.e., quantification of numerous other metabolites [32,41].

**Figure 2:**
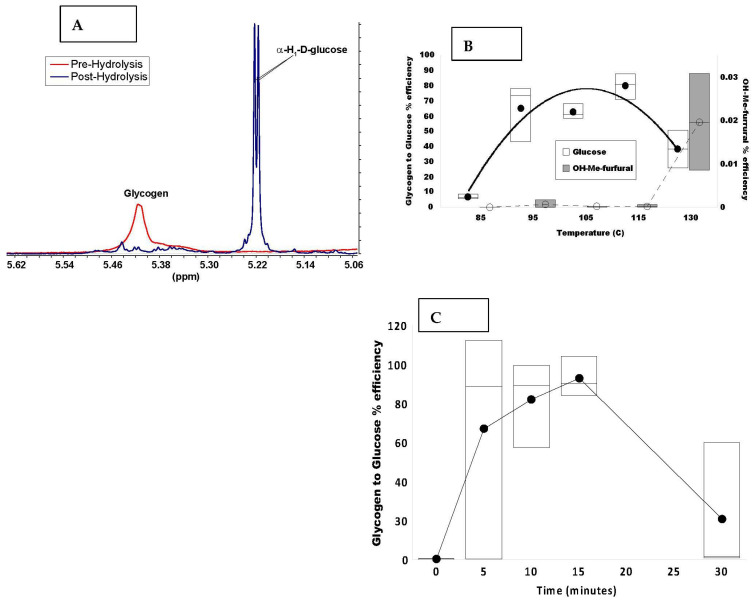
Dependence of the efficiency of glycogen acid hydrolysis to glucose on temperature and incubation time. The hydrolysis efficiency was determined as a function of temperature at a fixed hydrolysis time of 10 min and as a function of time at 110 °C using FT-MS and NMR as described in the Methods. (**A**). ^1^H NMR spectrum of glycogen pre (red) and post (blue) hydrolysis at 105 °C for 10 min. The region displayed includes the H1-α resonances of free glucose (5.22 ppm) and glycogen (5.42 ppm). (**B**). Left ordinate: The average glycogen to glucose hydrolysis efficiency (●) was determined as a function of temperature at a fixed hydrolysis time of 10 min. Open boxes represent quartiles. Right ordinate the average glycogen derived glucose oxidation to furfural (O) as a function of temperature (n = 3) with shaded boxes representing quartiles. These values are displaced +/− on the x-axis for clarity. (**C**). Hydrolysis efficiency at 110 °C versus time measured by UHR-FTMS (n = 3). Average (●) with quartiles.

We used purified bovine liver glycogen (Sigma) to optimize hydrolysis conditions in the CEM Discover microwave system described in the Methods. Preliminary testing indicated a reaction time of 10–15 min in 1 N HCl at 105 °C under nitrogen atmosphere effectively liberated glucose from glycogen. Figure 2A shows the anomeric region of the ^1^H NMR spectrum before and after 10 min of hydrolysis at 105 °C. Essentially all of the NMR-visible glycogen (broad resonance at 5.42 ppm) was hydrolyzed and at least 71% was recovered as free glucose (sharp doublet at 5.22 ppm). To establish the temperature dependence of the reaction, a solution of 0.5 mg glycogen in 30 µL 1 N HCl was incubated at the desired temperature for 15 min in nitrogen atmosphere. Maximal glucose recovery, as determined by direct infusion UHR-FTMS, occurred at 110 °C with a relatively broad plateau between ca. 105 °C and 115 °C (Figure 2B). In addition, we determined that the recovery of glucose at 110 °C was comparable between 5 and 15 min of hydrolysis but declined substantially by 30 min (Figure 2C). We have therefore chosen the conditions of 10 min heating at 105 or 110 °C for subsequent experiments.

We also measured the formation of hydroxymethylfurfural and levulinic acid as they have been reported to be major thermal degradation products of glucose [42]. Levulinic acid was detected at low amounts in non-hydrolyzed glycogen, and we found it to be an unreliable marker for temperature-dependent glucose degradation (data not shown). However, an inverse relationship between hydroxymethyl-furfural and glucose was evident above the peak glucose recovery temperature, which accounted for some of the observed drop in glucose recovery (Figure 2B).

Using the purified liver glycogen, we estimated the glucose recovery to be between 70% to 90% under the optimized conditions. The absolute recovery depended in part on the accuracy in weighing the original amount of glycogen and its reported purity in terms of glucose content. However, total quantitative uncertainty does not affect the determination of the highly informative ^13^C fractional enrichment in [^13^C_6_]-glucose tracer studies, as described below.

As cell or tissue samples contain large amounts of proteins amongst other components, we next tested the effects of proteins on the glycogen hydrolysis efficiency. An equivalent amount of BSA (0.5 mg) was added to the reaction mixture before hydrolysis. We observed a non-significant increase in optimal hydrolysis temperature from ca. 110 °C to ca. 115 °C, with no change in the optimal reaction time (data not shown). 

Having established the method for purified glycogen, we next applied the method to biological extracts derived from cell cultures, mouse tissues, and ex vivo human tissue cultures. 

### 3.2. Glycogen Analysis in Human Cells

The human primary lung BEAS-2B cells were grown in unlabeled glucose or ^13^C_6_-glucose tracer and extracted as described in the Methods. The extracted polar fraction was divided into 1 small fraction (1/16th volume) and 3 larger fractions (each approximately 1/3 of the remainder). The small polar fraction was used for pre-hydrolysis glucose measurement by UHR-FTMS, while the larger fractions were measured by NMR and UHR-FTMS pre- and post-hydrolysis. A standard addition curve was established for the UHR-FTMS method to measure the hydrolysis efficiency. Glycogen was calculated as stated in Section 2.

We analyzed the BEAS-2B cells grown in unlabeled glucose for hydrolyzed glycogen content. The combined average hydrolyzed glycogen content for BEAS-2B cells after correcting for hydrolytic efficiency was 37.5 ± 1.3 µg/mg protein based on the UHR-FTMS and NMR analysis (Table 1), which gave comparable glycogen content in µg/mg protein.

When grown in ^13^C_6_-glucose for 24 h, we found substantial ^13^C enrichment of intact glycogen in BEAS-2B cells, as determined by NMR. Figure S1 shows a ^1^H NMR (A) and corresponding 1D ^1^H{^13^C}HSQC (B) spectra of the anomeric region of a BEAS-2B cell extract. The ^13^C satellite resonances of the glycogen and free glucose + glucose-6-phosphate (Glc-6-P) in the ^1^H spectrum were prominent (Figure S1A), indicating high ^13^C enrichment. Using the peak areas, we determined the fraction of ^13^C to be 63% and 88% for glycogen and glucose + Glc-6-P, respectively.

**Figure 3:**
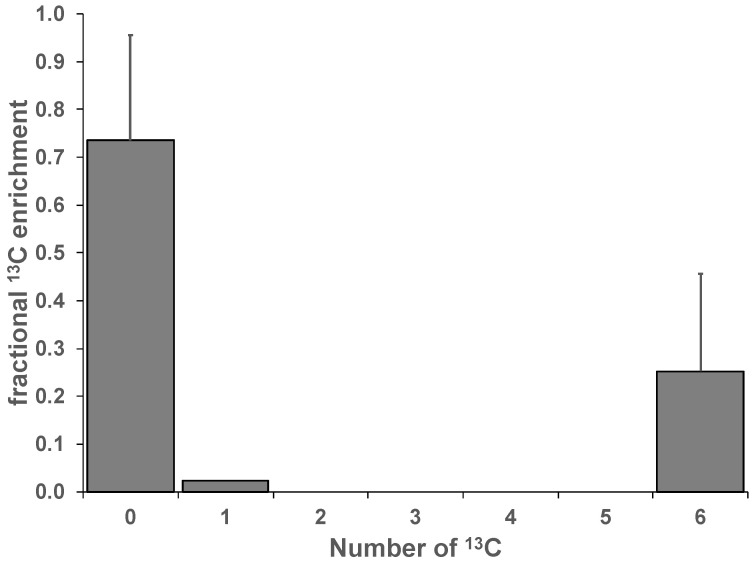
UHR-FTMS analysis of extractable glycogen in PBMC. Peripheral blood monocytic cells (PBMC) were isolated, differentiated, polarized, and cultured with ^13^C_6_-glucose for 24 h. Glycogen was extracted, hydrolyzed to glucose, and measured by UHR-FTMS, as described in the Methods (n = 2).

We also found substantial glycogen and ^13^C labeling in non-proliferative peripheral blood monocytic cell (PBMC)-derived human macrophages. After 24 h of incubation in ^13^C_6_-glucose, the NMR-visible glycogen, although at low abundance, was about 19% ^13^C enriched in M1 type human macrophages, which was accompanied by a buildup of the precursor ^13^C-UDP-glucose (UDPG) (Figure S2). Total glycogen after hydrolysis was calculated to be 790 ± 127 ng/mg protein with a ^13^C fractional enrichment of 25% essentially with only 0 or 6 ^13^C atoms, indicating minimal metabolic scrambling (Figure 3). Overall, these data show a wide range of glycogen content and apparent capacity of synthesis in different cell types.

### 3.3. Glycogen Analysis in Mouse Tissues

**Figure 4:**
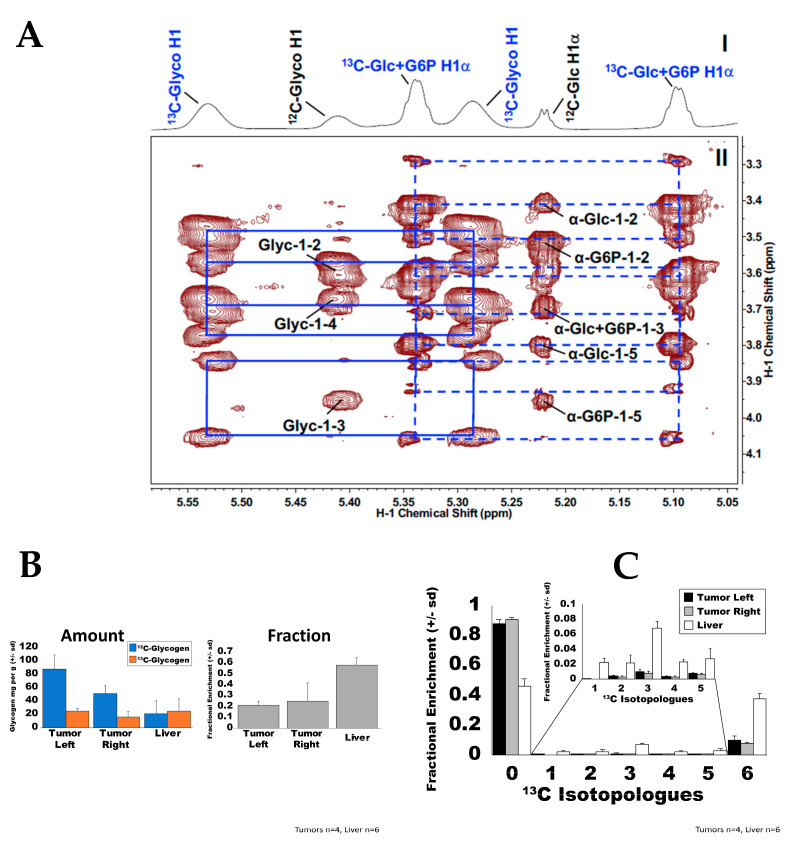
NMR and UHR-FTMS analysis of glycogen synthesis from ^13^C glucose in mouse liver extracts. C57BL/6 (**A**) or NSG mice bearing a human NSCLC xenograft (**B**,**C**) were fed a liquid diet containing 2.2 g [^13^C_6_]-glucose for 18 h, as described previously [16]. The mice were dissected for livers, which were extracted and analyzed by NMR and UHR-FTMS as described in the Methods. (**A**). 1D ^1^H (**I**) and TOCSY (**II**) spectra of a C57BL/6 mouse liver extract showing the anomeric region of glycogen (Glyc) and glucose + glucose-6-phosphate (Glc-6-P). Resonances were assigned based on 2D NMR of standards [35]. Solid and dashed boxes connect the ^13^C satellites of the cross peaks for glycogen and glucose + Glc-6-P, respectively. The ^13^C enrichment at H1 of glycogen was determined to be 80% and 85% for glucose and Glc-6-P by 1D NMR and confirmed by TOCSY. (**B**). NMR quantification of intact glycogen in the liver of NSG mice bearing a human NSCLC xenograft (n = 3). (**C**). UHR-FTMS analysis of fractional enrichment in the isotopologues of glucose derived from acid hydrolysis of the liver extracts from (**B**) (n = 3).

To apply the method to analyzing glycogen content and ^13^C labeling in vivo, we fed ^13^C_6_-glucose to two common mouse models, C57Bl/6 and NSG mice bearing patient derived tumor xenograft (PDTX), via liquid diet for 18 h as previously described [16]. The glucose content in tissue extracts pre- (Figure 4A(I)) and post-hydrolysis was determined by 1D ^1^H NMR for calculating the glycogen content, as described in Section 2. After correcting for glucose conversion efficiency, we determined the liver glycogen content of C57Bl/6 mice to be 264 ± 48 µg/mg protein (n = 3). This is comparable to levels reported for various strains of mice [25,43]. 

In addition, using 1D ^1^H and 2D ^1^H TOCSY NMR, ^12^C-/^13^C-glycogen and ^12^C-/^13^C-glucose + Glc-6-P can be readily discerned by the ^1^H resonances at different positions (Figure 4A(II)). This allows for ^13^C positional isotopomer analysis for glycogen in C57Bl/6J mouse liver extracts prior to acid hydrolysis. The resonances at 5.41 and 5.22 ppm arose from the H1α attached to ^12^C1 of glycogen and glucose + Glc-6-P, respectively, while the broad peaks at 5.53/5.28 and 5.34/5.10 ppm were the corresponding ^13^C satellite resonances. Both glucose and glycogen were highly ^13^C enriched and to the same extent (86 ± 2% for glycogen and 85 ± 2% for glucose + Glc-6-P) at the C1 position as measured from the 1D spectrum (Figure 4A(I)). However, the 1D spectrum was not suited for determining the ^13^C enrichment at C2-4 as the ^13^C satellite resonances for these positions were not resolved. On the other hand, they were sufficiently resolved in the 2D spectrum, as illustrated by the separation of the ^13^C satellite cross-peaks for H1 to H2, H3, or H4 (Figure 4A(II) and data not shown). The fractional enrichment at these glycogen and glucose positions was obtained by volume integration of the appropriate cross-peaks as previously described [36,44], which were 87 ± 3%, 90 ± 4% and 85 ± 2% for C2, C3, and C4 of glycogen, compared with 80 ± 4% and 88 ± 3% for C2 and C5 of glucose, respectively. These were similar to the % ^13^C enrichment for C1 of glycogen and glucose + Glc-6-P determined from the 1D spectrum. Together, these results point to a predominant amount of direct ^13^C_6_-glucose incorporation into NMR-visible glycogen without significant ^13^C scrambling. 

Prior to acid hydrolysis, the total NMR-visible glycogen content of liver from an NSG mouse bearing human patient-derived lung cancer xenograft (PDTX) was estimated at 38.3 ± 6.9 µg/mg residue weight (n = 6) based on ^1^H NMR analysis (Figure 4B; Table S1). The ^12^C-glycogen content was measured to be 21.1 ± 0.8 µg/mg protein residue weight (54.9 ± 0.3%) and ^13^C glycogen as 17.3 ± 0.6 µg/mg protein residue weight (45.1 ± 0.3%). 

After hydrolysis, the calculated total liver glycogen for the PDTX bearing NSG mouse was 44.8 ± 7.5 µg/mg pellet weight (n = 6). The ^12^C- glycogen content was determined to be 20.4 ± 3.9 µg/mg protein residue weight (42 ± 1.4%) and ^13^C glycogen as 24.3 ± 3.7 µg/mg protein residue weight (58 ± 1.4%) This degree of enrichment is comparable to that reported by Grutter et al. [45], who found 57% of liver glycogen in humans is newly synthesized in a fed state. In support of the NMR analysis, the UHR-FTMS analysis of the corresponding acid-hydrolyzed liver extracts showed that 46.3 ± 0.9% of the glucose was unlabeled (Figure 4C). 

In contrast to the liver, the PDTX tumors had higher pre-hydrolysis NMR-visible glycogen (63.6 ± 5.8 and 72.0 ± 3.1 mg/g protein residue weight, for the right and left tumor, respectively). However, PDTX tumors had a significantly lower ^13^C fractional enrichment (19.7/22.0 ± 0.6/0.5% and 24.7/21.3 ± 1.9/0.9% tumor right/left) when compared to the liver (*p* < 0.001) (Supplementary Material Table S1).

The UHR-FTMS analysis of the corresponding acid-hydrolyzed extracts showed similar enrichment patterns to the NMR-visible glycogen data. (Figure 4C). The PDTX tumors were largely composed of unlabeled glucose (87.4 ± 0.9% and 90.4 ± 0.6% for the left and right tumors respectively). The liver was 46.3 ± 0.9% ^12^C_6_, 37.6 ± 0.7% ^13^C_6_ and 16.1 ± 0.8% ^13^C scrambled as ^13^C_1_ to ^13^C_5_ isotopologues (Figure 4C and Figure S3); ^13^C scrambling can result from gluconeogenic and/or the pentose phosphate pathway (PPP) activity. The liver, the major gluconeogenic tissue, showed much higher levels of the scrambled isotopologues than in the tumors, though a more detailed analysis would require detailed quantitative modeling.

Moreover, the values obtained for NSG mice differed from those obtained for C57Bl/6J mice, which may reflect strain differences and/or the influence of tumor growth on liver glycogen metabolism. 

### 3.4. Glycogen Analysis in Human Ex Vivo Tissue Slices

**Figure 5:**
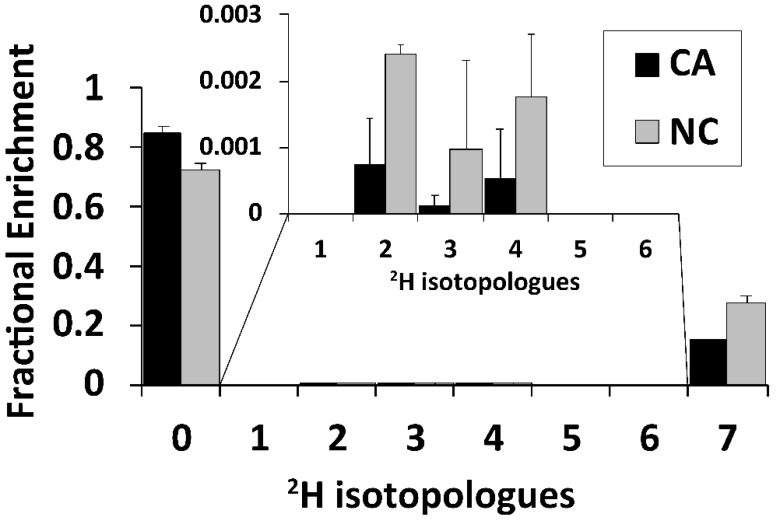
UHR-FTMS analysis of glycogen in ex vivo lung OTCs treated with triple tracers. Cancerous (CA) and non-cancerous (NC) tissues from an NSCLC patient (UK144) were sliced (n = 2), incubated in ^2^H_7_-Glucose, ^13^C_5_-glutamine, and ^15^N_2_-tryptophan for 48 h, extracted, and analyzed by UHR-FTMS for glycogen via acid hydrolysis as described in the Methods. CA OTCs had a greater amount of total glycogen compared to the matched NC OTCs (41.0 versus 14.1 µg/mg protein) (Figure S4) but the fractional enrichment of newly added D-glucose in glycogen was much higher in NC OTCs compared to the CA OTCs. Inset shows the distribution of scrambled ^2^H_1_- to ^2^H_6_-isotopologues of glucose (n = 2).

We further demonstrated the utility of the method in determining the glycogen content of ex vivo human lung OTCs [14,31,33]). Cancerous (CA) and surrounding non-cancerous (NC) lung tissues were surgically resected from an NSCLC patient. A part of the tissue was snap-frozen as bulk tissue and the rest was thinly sliced and incubated for 48 h in a triple tracer medium containing ^2^H_7_-Glucose, ^13^C_5_-glutamine, and ^15^N_2_-tryptophan before processing and extraction for polar metabolites as described previously [31] and in the Methods. The glucose isotopologue composition of glycogen in µg/mg protein was determined from the pre- and post-acid hydrolyzed extracts by direct-infusion UHR-FTMS as shown in Figure S4. The CA OTCs showed large variations between slices but on average contained more total glycogen (43.3 ± 25.3 μg/mg) than the NC tissue slices (21.3± 5.3 μg/mg). This difference is similar to what was observed for the glycogen content of the corresponding bulk tissues that were immediately frozen after resection from the patient (Figure S5). However, the level of the dominant labeled isotopologue (^2^H_7_-glycogen) was comparable for the CA (6.3 ± 2.7) and NC (5.9 ± 2.0 µg/mg protein) OTCs (Figure S4). This resulted in a higher fractional enrichment of ^2^H_7_-glycogen in the NC than in the CA OTC (27% versus 15%) (Figure 5), which suggests higher glycogen turnover in the NC than its matched CA lung tissue. Moreover, presence of ^2^H_2_ to ^2^H_4_ isotopologues is consistent with scrambling processes, as noted for the ^13^C_1_ to ^13^C_5_ isotopologues of glycogen-derived glucose for PDX tumors (cf. Figure 4). Although the extent of scrambling was low, it appeared to be greater in the NC lung tissue than in the CA tissue.

In these triple tracer experiments, we also noted the lack of detectable ^13^C_5_-Gln incorporation into glycogen in either CA or NC lung OTCs, which indicates no significant gluconeogenesis from Gln in these tissues under these conditions. However, we did observe a small amount of scrambled ^2^H labeled glycogen (^2^H_2_ to ^2^H_4_-isotopologues, Figure 5 and Figure S4), which can be attributed to the PPP activity as gluconeogenic activity was absent. Thus, the expanded pathway coverage afforded by combining multiplex SIRM (mSIRM) [27] with our new glycogen analytical method verified glycogen synthesis from glucose directly and via PPP but not from Gln via gluconeogenesis. This level of detail cannot be ascertained with the single tracer-based SIRM approach described above for the mouse PDTX model (Figure 4C) and without the capability of UHR-FTMS and NMR for resolving multiple tracer atoms in glycogen and related metabolites such as the precursor UDP-glucose (UDPG) (Figure S1 and Figure S2). It should also be noted that the mSIRM approach eliminated the confounding influence of lung CA tissue heterogeneities (cf. Figure 5) on delineating the different paths of glycogen synthesis and their dynamics since they were tracked by different tracers in the same OTC as opposed to tracking by a single tracer in separate OTCs. A further added advantage was the reduced demand for biological samples, which is particularly important for studies with limited sample availability, e.g., in the case of human biospecimens.

## 4. Discussion

Glycogen is present in many tissues and its metabolism is highly regulated in mammalian systems. As dysregulation of glycogen metabolism occurs in human cancer and other diseases, the ability to determine glycogen content and turnover using very small quantities of patient specimens with the capacity for atom-resolved tracing is critically important for understanding the role of glycogen metabolism in the development and progression of human diseases. We have described a very sensitive (fmol in glucose equivalents) method for analyzing glycogen and its turnover that is applicable to a wide range of biological systems and is compatible with mSIRM analysis by NMR and UHR-FTMS using very small amounts of biological materials. 

We have demonstrated the method on widely different specimens ranging from human lung cells/macrophages, mouse liver, to human CA and NC lung tissues and OTCs. We found that NMR-detected glycogen in the NSG mouse was about 64% and 85% of the total glycogen (as determined by hydrolysis) in the left PDX tumor and liver respectively (Table S1). These imply glycogen particles with a sizable immobile core in addition to mobile solvent-exposed branches that are visible by NMR in both tissue types. We also saw lower ^13^C fractional enrichment in the NMR-determined intact (pre-hydrolysis values) than total glycogen (post-hydrolysis values) for the liver and right PDX tumor but not for the left PDX tumor (Table S1), suggesting preferential degradation of newly synthesized, NMR-detected glycogen in the former two cases. Moreover, the distinct data on PDX tumors point to heterogeneities in the glycogen metabolism machinery in the starting pieces of patient-derived tumor tissues for the xenograft and/or differences in the xenograft environment of the mouse host. 

In addition, the use of multiple tracers in the lung OTC studies illustrates the value of coupling this method with mSIRM in resolving the contribution of different pathways to glycogen synthesis, i.e., synthesis directly using supplied glucose (D_7_-glucose), PPP-derived glucose (D-scrambled glucose), or gluconeogenesis-derived glucose (^13^C_5_-Gln-derived glucose). Thus, this combination has the capacity for resolving more routes of glycogen synthesis with more rigor than those afforded by non-tracer or even single tracer studies, while greatly reducing the requirement (mg or biopsy level) for samples and eliminating the influence of tissue heterogeneity or sample batch variation on pathway interpretation. It should also be noted that the method is readily integrated with existing SIRM workflows, allowing for the tracing of glycogen turnover along with many other metabolic pathways to be achieved in single extracts (e.g., [14,33,44]) without the need for additional experiments. 

Moreover, this method is highly amenable for automation to increase sample throughput and reduce labor. We have already semi-automated the workflow by batch-processing the microwave assisted acid hydrolysis and UHR-FTMS analysis, which is capable of processing ≥ 120 samples per day. 

## 5. Conclusions

We have established a microscale quantitative method for intact and acid-hydrolyzed glycogen determination by combining NMR with UHR-FTMS analysis, which offers important benefits over existing methods. These include a much-reduced sample requirement, higher sample throughput with reduced labor, full compatibility with mSIRM workflows for expanded and rigorous coverage of glycogen metabolism and many other metabolic pathways, as well as elimination of the influence of tissue heterogeneity or sample batches on pathway interpretation. The versatility of this method was demonstrated in multiple sample types and their tracer studies, including primary human cell lines, differentiated human macrophages, and human/mouse tissues. By tracing the fate of D_7_-glucose + ^13^C_5_-Gln, we ascertained the major route of glycogen synthesis as direct glucose incorporation with the minor incorporation of glucose derived from PPP but not from gluconeogenesis in patient-derived lung tissues, along with reduced glycogen turnover in cancerous tissues versus matched non-cancerous counterparts. This knowledge would be difficult to attain without this newly developed method.

## Figures and Tables

**Table 1:** NMR and FTMS analysis of extractable glycogen in BEAS-2B cells.

Compound	Average ^2^ n = 4	FTMS n = 2	NMR H1β ^3^ n = 2	NMR H1α ^3^ n = 2
Pre-Hydrolysis Glucose (nmol) ^1^	29.1 ± 2.4	29.1 ± 2.4	<LoQ ^4^	<LoQ
Post-Hydrolysis Glucose (nmol) ^1^	121.1 ± 18.0	140.5 ± 25.5	105.0 ± 17.9	117.9 ± 21.7
Average Glycogen (µg) ^1^	16.9 ± 1.1	18.0 ± 3.7	15.9 ± 3.1	16.7 ± 4.5
Average Hydrolysis Efficiency (µg)	90.8 ± 14.1	74.5 ± 9.8	97.8 ± 12.1	100 ± 16.3
Corrected Glycogen (µg)	22.7 ± 1.4	24.2 ± 5.0	21.4 ± 4.1	22.4 ± 6.1
Glycogen per mg protein (µg/mg)	37.5 ± 1.3	39.7 ± 6.7	34.7 ± 6.6	38.1 ± 3.6

^1^ Values shown are means and sem; ^2^ average of FTMS and NMR data combined; **^3^** H1β, H1α are the two anomeric forms of glucose; ^4^ LoQ limit of quantification.

## Data Availability

The datasets used and/or analyzed for the current study are available from the corresponding authors on reasonable request. The data are not publicly available due to lack of a suitable repository.

## Supplementary Materials

The following supporting information can be downloaded at: https://www.mdpi.com/article/10.3390/metabo12080760/s1, Table S1: NMR analysis of Pre- and Post-hydrolysis NSG mouse xenograft tissue glycogen; Figure S1: ^1^H and HSQC NMR spectra of BEAS-2B cells grown in ^13^C_6_-glucose; Figure S2: 1D HSQC NMR spectra of M1 macrophages grown in ^13^C_6_-glucose; Figure S3: UHR-FTMS spectra of liver extract of a PDTX-NSG mouse fed ^13^C_6_-glucose; Figure S4: UHR-FTMS analysis of ^2^H fractional enrichment of glycogen in cancerous and non-cancerous lung OTCs; Figure S5. UHR-FTMS analysis of glycogen in bulk cancerous and non-Cancerous lung tissues.

## References

[1] Ipata P.L., Balestri F. (2012). Glycogen as a fuel: Metabolic interaction between glycogen and ATP catabolism in oxygen-independent muscle contraction. Metabolomics.

[2] Favaro E., Bensaad K., Chong M.G., Tennant D.A., Ferguson D.J., Snell C., Steers G., Turley H., Li J.L., Gunther U.L. (2012). Glucose utilization via glycogen phosphorylase sustains proliferation and prevents premature senescence in cancer cells. Cell Metab..

[3] Hicks J., Wartchow E., Mierau G. (2011). Glycogen storage diseases: A brief review and update on clinical features, genetic abnormalities, pathologic features, and treatment. Ultrastruct. Pathol..

[4] Rousset M., Zweibaum A., Fogh J. (1981). Presence of Glycogen and Growth-related Variations in 58 Cultured Human Tumor Cell Lines of Various Tissue Origins. Cancer Res..

[5] Zois C.E., Harris A.L. (2016). Glycogen metabolism has a key role in the cancer microenvironment and provides new targets for cancer therapy. J. Mol. Med..

[6] Adeva-Andany M.M., Gonzalez-Lucan M., Donapetry-Garcia C., Fernandez-Fernandez C., Ameneiros-Rodriguez E. (2016). Glycogen metabolism in humans. BBA Clin..

[7] Whelan W.J. (1986). The initiation of glycogen synthesis. Bioessays.

[8] Lomako J., Lomako W.M., Whelan W.J. (2004). Glycogenin: The primer for mammalian and yeast glycogen synthesis. Biochim. Biophys. Acta.

[9] Radziuk J., Pye S. (2001). Hepatic glucose uptake, gluconeogenesis and the regulation of glycogen synthesis. Diabetes Metab. Res. Rev..

[10] Bouskila M., Hunter R.W., Ibrahim A.F., Delattre L., Peggie M., van Diepen J.A., Voshol P.J., Jensen J., Sakamoto K. (2010). Allosteric regulation of glycogen synthase controls glycogen synthesis in muscle. Cell Metab..

[11] Mathieu C., Bui L.C., Petit E., Haddad I., Agbulut O., Vinh J., Dupret J.M., Rodrigues-Lima F. (2017). Molecular Mechanisms of Allosteric Inhibition of Brain Glycogen Phosphorylase by Neurotoxic Dithiocarbamate Chemicals. J. Biol. Chem..

[12] Zois C.E., Favaro E., Harris A.L. (2014). Glycogen metabolism in cancer. Biochem. Pharm..

[13] Fan T.W., Lane A.N., Higashi R.M., Farag M.A., Gao H., Bousamra M., Miller D.M. (2009). Altered regulation of metabolic pathways in human lung cancer discerned by (13)C stable isotope-resolved metabolomics (SIRM). Mol. Cancer.

[14] Sellers K., Fox M.P., Bousamra M., Slone S.P., Higashi R.M., Miller D.M., Wang Y., Yan J., Yuneva M.O., Deshpande R. (2015). Pyruvate carboxylase is critical for non-small-cell lung cancer proliferation. J. Clin. Investig..

[15] Winnike J., Pediaditakis P., Wolak J., McClelland R., Watkins P., Macdonald J. (2012). Stable isotope resolved metabolomics of primary human hepatocytes reveals a stressed phenotype. Metabolomics.

[16] Sun R.C., Fan T.W., Deng P., Higashi R.M., Lane A.N., Le A.T., Scott T.L., Sun Q., Warmoes M.O., Yang Y. (2017). Noninvasive liquid diet delivery of stable isotopes into mouse models for deep metabolic network tracing. Nat. Commun..

[17] Revel J.P., Napolitano L., Fawcett D.W. (1960). Identification of glycogen in electron micrographs of thin tissue sections. J. Biophys. Biochem. Cytol..

[18] Baba O. (1993). Production of Monoclonal Antibody That Recognizes Glycogen and Its Application for Immunohistochemistry. J. Stomatol. Soc..

[19] Cifuentes D., Martínez-Pons C., García-Rocha M., Galina A., De Pouplana L.R., Guinovart J.J. (2008). Hepatic glycogen synthesis in the absence of glucokinase: The case of embryonic liver. J. Biol. Chem..

[20] Puri R., Jain N., Ganesh S. (2011). Increased glucose concentration results in reduced proteasomal activity and the formation of glycogen positive aggresomal structures. FEBS J..

[21] Prats C., Gomez-Cabello A., Nordby P., Andersen J.L., Helge J.W., Dela F., Baba O., Ploug T. (2013). An Optimized Histochemical Method to Assess Skeletal Muscle Glycogen and Lipid Stores Reveals Two Metabolically Distinct Populations of Type I Muscle Fibers. PLoS ONE.

[22] Mc M.J. (1948). Histological and histochemical uses of periodic acid. Stain Technol..

[23] Louzao M.C., Espina B., Vieytes M.R., Vega F.V., Rubiolo J.A., Baba O., Terashima T., Botana L.M. (2008). “Fluorescent glycogen” formation with sensibility for in vivo and in vitro detection. Glycoconj. J..

[24] Witney T.H., Carroll L., Alam I.S., Chandrashekran A., Nguyen Q.-D., Sala R., Harris R., DeBerardinis R.J., Agarwal R., Aboagye E.O. (2014). A Novel Radiotracer to Image Glycogen Metabolism in Tumors by Positron Emission Tomography. Cancer Res..

[25] Passonneau J.V., Lauderdale V.R. (1974). A comparison of three methods of glycogen measurement in tissues. Anal. Biochem..

[26] Sun R.C., Dukhande V.V., Zhou Z., Young L.E.A., Emanuelle S., Brainson C.F., Gentry M.S. (2019). Nuclear Glycogenolysis Modulates Histone Acetylation in Human Non-Small Cell Lung Cancers. Cell Metab..

[27] Yang Y., Fan T.W., Lane A.N., Higashi R.M. (2017). Chloroformate derivatization for tracing the fate of Amino acids in cells and tissues by multiple stable isotope resolved metabolomics (mSIRM). Anal. Chim. Acta.

[28] Lane A.N., Fan T.W. (2017). NMR-based Stable Isotope Resolved Metabolomics in systems biochemistry. Arch. Biochem. Biophys..

[29] Fan T.W., Lane A.N. (2016). Applications of NMR spectroscopy to systems biochemistry. Prog. Nucl. Magn. Reson. Spectrosc..

[30] Bruntz R.C., Lane A.N., Higashi R.M., Fan T.W. (2017). Exploring cancer metabolism using stable isotope-resolved metabolomics (SIRM). J. Biol. Chem..

[31] Fan T.W., Lane A.N., Higashi R.M. (2016). Stable Isotope Resolved Metabolomics Studies in Ex Vivo TIssue Slices. Bio. Protoc..

[32] Fan T.W., Lorkiewicz P.K., Sellers K., Moseley H.N., Higashi R.M., Lane A.N. (2012). Stable isotope-resolved metabolomics and applications for drug development. Pharmacol. Ther..

[33] Fan T.W., Warmoes M.O., Sun Q., Song H., Turchan-Cholewo J., Martin J.T., Mahan A., Higashi R.M., Lane A.N. (2016). Distinctly perturbed metabolic networks underlie differential tumor tissue damages induced by immune modulator beta-glucan in a two-case ex vivo non-small-cell lung cancer study. Cold Spring Harb. Mol. Case Stud..

[34] Zang L.H., Rothman D.L., Shulman R.G. (1990). 1H NMR visibility of mammalian glycogen in solution. Proc. Natl. Acad. Sci. USA.

[35] Fan T.W., Lane A.N., Lutz N., Sweedler J.V., Weevers R.A. (2013). Assignment strategies for NMR resonances in metabolomics research. Methodologies for Metabolomics.

[36] Fan T.W.M., Lane A.N. (2008). Structure-based profiling of metabolites and isotopomers by NMR. Prog. Nucl. Magn. Reson. Spectrosc..

[37] Heinicke K., Dimitrov I.E., Romain N., Cheshkov S., Ren J., Malloy C.R., Haller R.G. (2014). Reproducibility and absolute quantification of muscle glycogen in patients with glycogen storage disease by 13C NMR spectroscopy at 7 Tesla. PLoS ONE.

[38] Passonneau J.V., Gatfield P.D., Schulz D.W., Lowry O.H. (1967). An enzymic method for measurement of glycogen. Anal. Biochem..

[39] Montgomery R. (1957). Determination glycogen. Arch. Biochem. Biophys..

[40] Hultman E. (1967). Muscle glycogen in man determined in needle biopsy specimens: Method and normal values. Scand. J. Clin. Lab. Investig..

[41] Fan T.W.M., Bruntz R.C., Yang Y., Song H., Chernyavskaya Y., Deng P., Zhang Y., Shah P.P., Beverly L.J., Qi Z. (2019). De novo synthesis of serine and glycine fuels purine nucleotide biosynthesis in human lung cancer tissues. J. Biol. Chem..

[42] Klein M., Pulidindi I.N., Perkas N., Meltzer-Mats E., Gruzman A., Gedanken A. (2012). Direct production of glucose from glycogen under microwave irradiation. RSC Adv..

[43] Shull K.H., Mayer J. (1956). The turnover of liver glycogen in obese hyperglycemic mice. J. Biol. Chem..

[44] Lane A.N., Fan T.W.M. (2007). Quantification and identification of isotopomer distributions of metabolites in crude cell extracts using 1H TOCSY. Metabolomics.

[45] Gruetter R., Magnusson I., Rothman D.L., Avison M.J., Shulman R.G., Shulman G.I. (1994). Validation of 13C NMR measurements of liver glycogen in vivo. Magn. Reson. Med..

