# Supplementary material for: A Micro-Scale Analytical Method for Determining Glycogen Turnover by NMR and FTMS
Source: Metabolites. 2022 Aug 18;12(8):760. doi: 10.3390/metabo12080760 (PMC9415681; doi:10.3390/metabo12080760)

Supplementary Materials

Table S1. NMR analysis of Pre- and Post-hydrolysis NSG mouse xenograft tissue glycogen

	**^{12}C**	**^{13}C**	**total**	**% ^{13}C**
**Liver (n=6)**				
Pre-hydrolysis (mg / g residue equivalent)	21.1 ± 0.8	17.3 ± 0.6	38.3 ± 1.4	45.1% ± 0.3%
Post-hydrolysis* (mg / g residue)	20.4 ± 3.9	24.3 ± 3.7	44.8 ± 7.5	58.0% ± 1.4%
**Tumor Right (n=4)**				
Pre-hydrolysis (mg / g residue equivalent)	50.8 ± 4.4	12.7 ± 1.4	63.6 ± 5.8	19.7% ± 0.6%
Post-hydrolysis* (mg / g residue)	50.7 ± 4.7	16.0 ± 0.5	66.7 ± 4.9	24.7% ± 1.9%
**Tumor Left (n=4)**				
Pre-hydrolysis (mg / g residue equivalent)	56.2 ± 2.4	15.9 ± 0.8	72.0 ± 3.1	22.0% ± 0.3%
Post-hydrolysis* (mg / g residue)	88.2 ± 3.9	24.4 ± 7.5	112.6 ± 6.4	21.3% ± 0.6%

* calculated, background subtracted, and hydrolysis efficiency corrected, SEM.

Figure S1. ^{1}H and HSQC NMR spectra of BEAS-2B cells grown in $^{13}C_6$-glucose. BEAS-2B cells were incubated in $^{13}C_6$-glucose for 24 h before extraction for polar metabolites and NMR analysis as described in the Method. Panels **A** and **B** show respectively 1D ^{1}H and ^{1}H{^{13}C}HSQC spectra in the anomeric region of glycogen and glucose (Glc)+glucose-6-phosphate (Glc-6-P) for the polar extracts. Free glucose+Glc-6-P was estimated to be 68% ^{13}C enriched, compared to 60% ^{13}C enrichment for the NMR-visible glycogen.

.

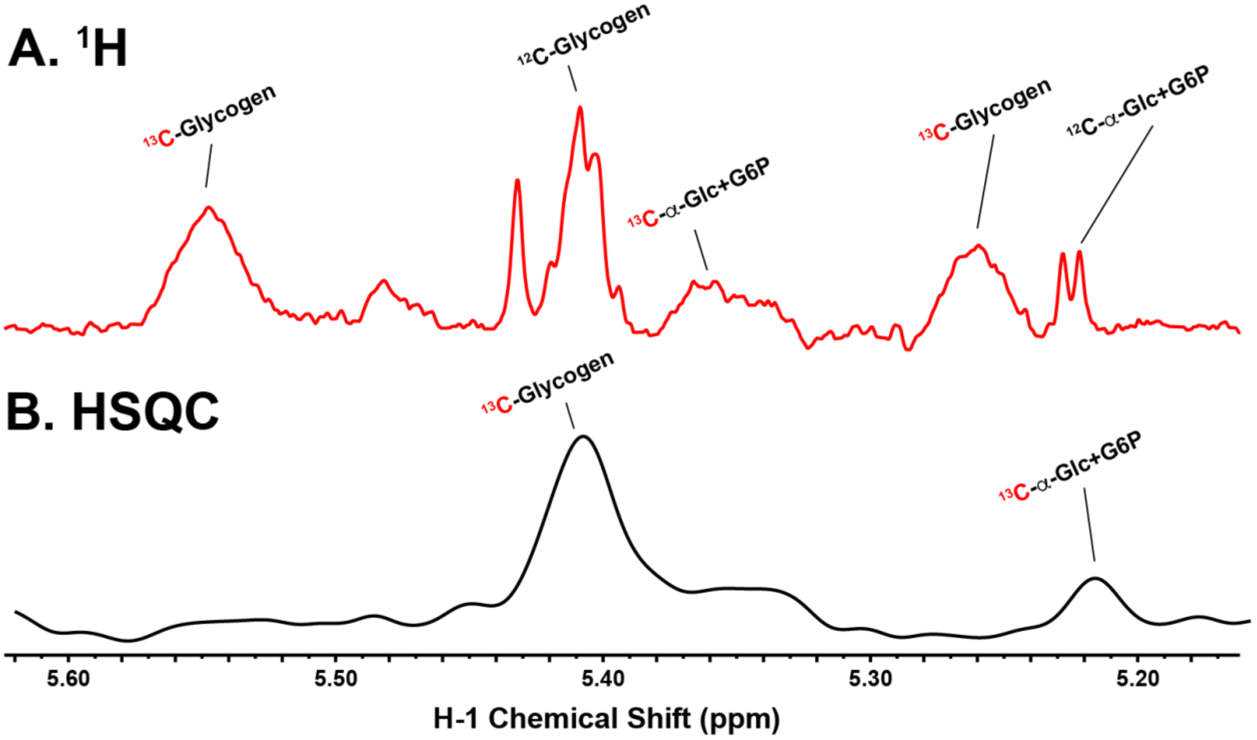

Figure S2. 1D $^{1}H\{^{13}C\}$-HSQC NMR spectra of M1 macrophages grown in $^{13}C_6$-glucose.

M1 polarized macrophages were grown in $^{13}C_6$-glucose, extracted for polar metabolites, and analyzed by NMR as described in the Methods. The 1D $^{1}H\{^{13}C\}$HSQC spectrum in the anomeric region showed ^{13}C labeling in glucose+Glc-6-P at C1, at the C1 of the glucose subunit UDP-glucose (glycogen precursor), UDP-galactose and UDPGlcNAc. The total glycogen content and ^{13}C labeling was much lower in these cells than in BEAS-2B cells (cf Fig. S1).

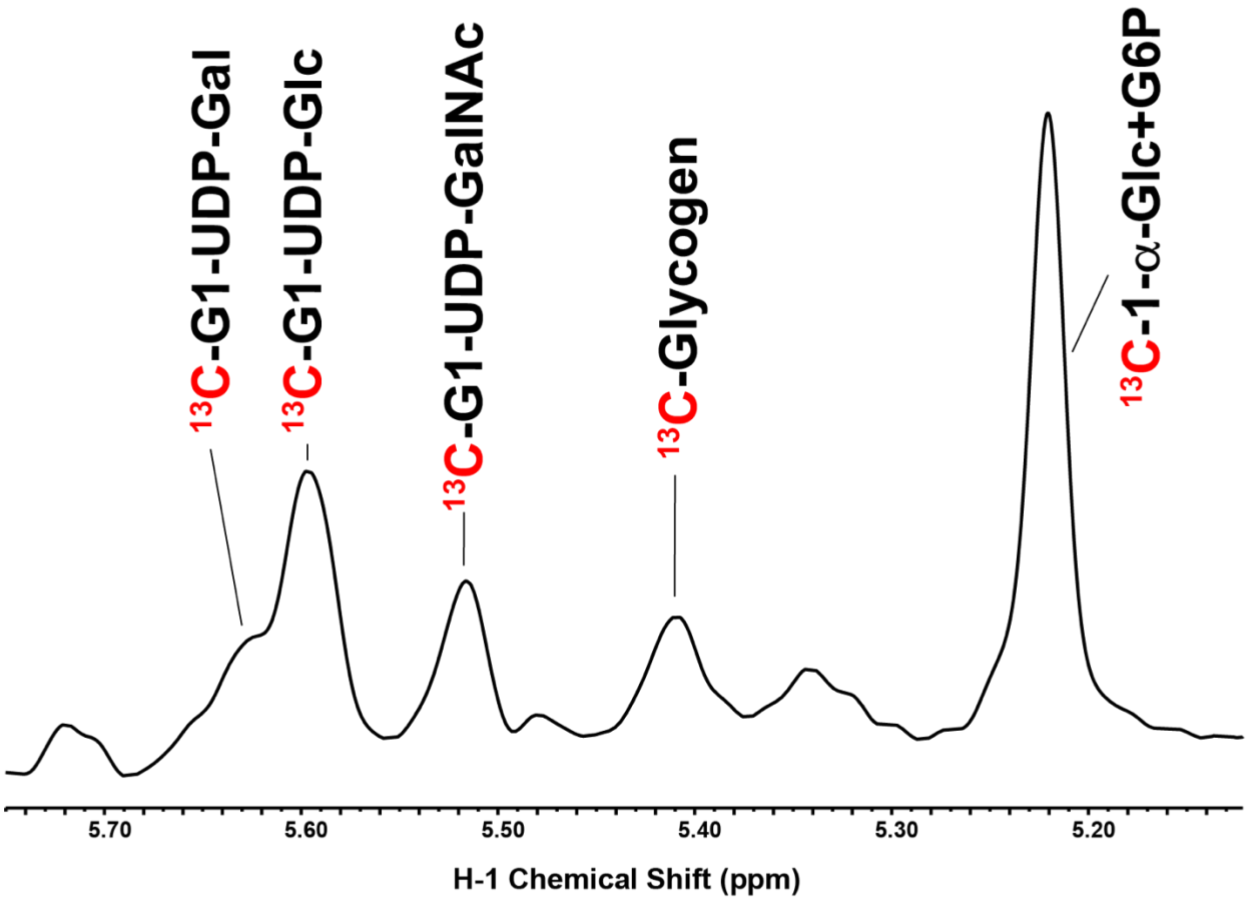

Figure S3. UHR-FTMS spectra of liver extract of a PDTX-NSG mouse fed $^{13}C_6$-glucose.

A representative UHR-FTMS spectrum of the acid-hydrolyzed glucose for extracts in **Fig. 4C** showed the isotopologue distribution with $^{13}C_6$, $^{2}H_7$-glucose as a calibration and quantification standard. Inset shows a magnified region of the $^{13}C_1$- through $^{13}C_6$-isotopologues of glucose.

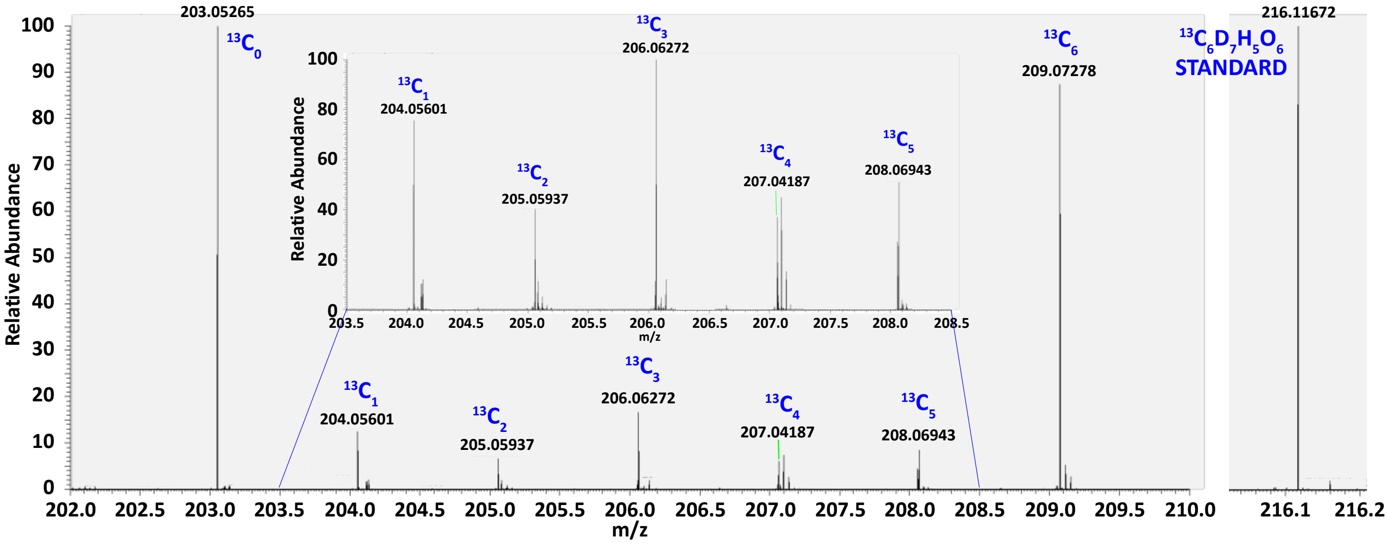

Figure S4. UHR-FTMS analysis of ^{2}H fractional enrichment of glycogen in cancerous and non-cancerous lung tissue slice cultures.

Glycogen was quantified post-hydrolysis. Total glycogen in the CA OTCs (43.3 ± 25.4 µg/mg protein, n=2) was higher than the NC (21.3 ± 5.4 µg/mg protein, n=2). However, newly synthesized (1-7 or $^2H_1$-$^2H_7$) glycogen comprised a greater fraction of total glycogen in the NC than CA OTCs (cf. **Fig. 5**). As observed for liver (Fig 5), though at a lower level, other isotopologues were observed consistent with metabolic scrambling via the pentose phosphate pathway and/or gluconeogenesis.

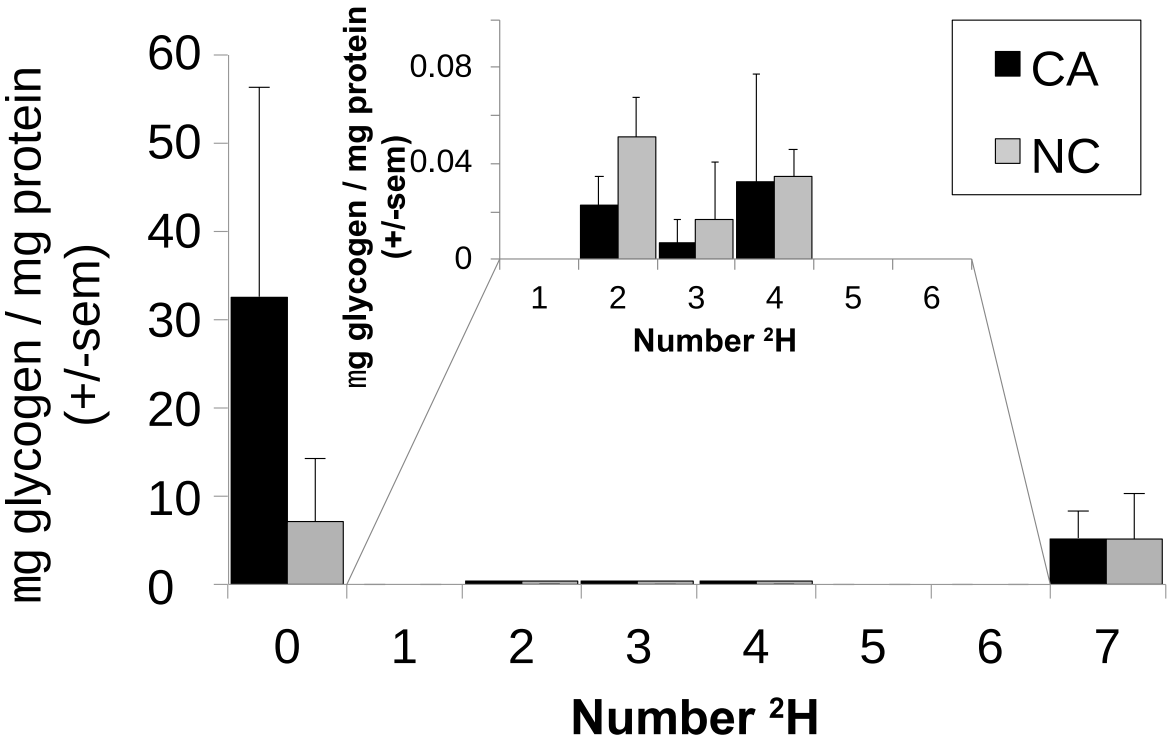

Figure S5. UHR-FTMS analysis of glycogen in bulk cancerous and non-cancerous lung tissues.

Within minutes of excision from a NSCLC patient a small portion of cancerous (CA) or non-cancerous (NC) bulk lung tissue was immediately flash-frozen in liquid nitrogen, processed, and extracted for polar metabolites as described in the Methods. Additional tissues were sliced and used for SIRM experiments in (Fig. 5) as described in the Methods. Total glycogen content was calculated as described in the Methods. CA and NC tissues had respectively 33.7 and 12.3 µg glycogen/mg protein (n=1).

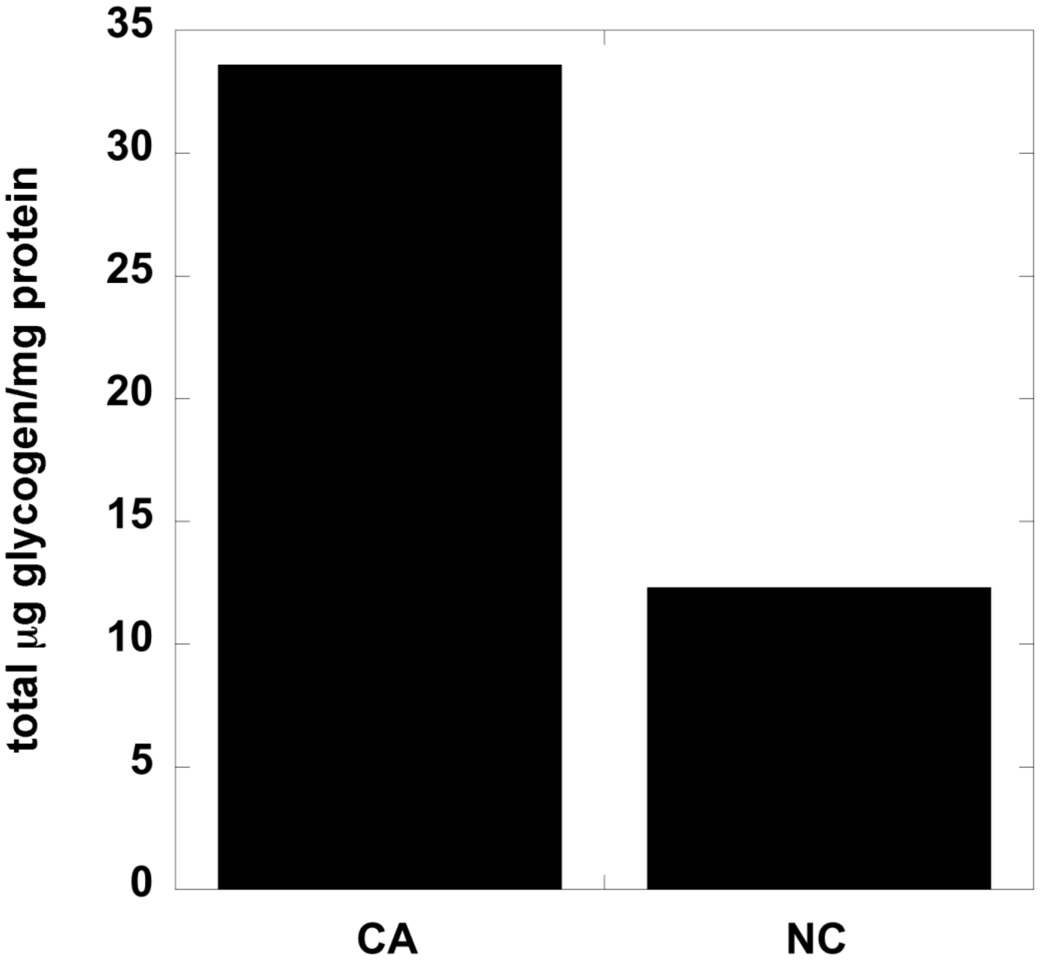

Supplement: Supplementary file 1 [file metabolites-12-00760-s001.zip › metabolites-1831188-supplementary.pdf]
